# An Automated Indoor Localization System for Online Bluetooth Signal Strength Modeling Using Visual-Inertial SLAM

**DOI:** 10.3390/s21082857

**Published:** 2021-04-19

**Authors:** Simon Tomažič, Igor Škrjanc

**Affiliations:** Faculty of Electrical Engineering, University of Ljubljana, 1000 Ljubljana, Slovenia; igor.skrjanc@fe.uni-lj.si

**Keywords:** indoor localization, visual-inertial SLAM, constrained optimization, path loss model, particle swarm optimization, Bluetooth low energy, beacon

## Abstract

Indoor localization is becoming increasingly important but is not yet widespread because installing the necessary infrastructure is often time-consuming and labor-intensive, which drives up the price. This paper presents an automated indoor localization system that combines all the necessary components to realize low-cost Bluetooth localization with the least data acquisition and network configuration overhead. The proposed system incorporates a sophisticated visual-inertial localization algorithm for a fully automated collection of Bluetooth signal strength data. A suitable collection of measurements can be quickly and easily performed, clearly defining which part of the space is not yet well covered by measurements. The obtained measurements, which can also be collected via the crowdsourcing approach, are used within a constrained nonlinear optimization algorithm. The latter is implemented on a smartphone and allows the online determination of the beacons’ locations and the construction of path loss models, which are validated in real-time using the particle swarm localization algorithm. The proposed system represents an advanced innovation as the application user can quickly find out when there are enough data collected for the expected radiolocation accuracy. In this way, radiolocation becomes much less time-consuming and labor-intensive as the configuration time is reduced by more than half. The experiment results show that the proposed system achieves a good trade-off in terms of network setup complexity and localization accuracy. The developed system for automated data acquisition and online modeling on a smartphone has proved to be very useful, as it can significantly simplify and speed up the installation of the Bluetooth network, especially in wide-area facilities.

## 1. Introduction

Recently, the social and commercial interest in Location-Based Services (LBS) is increasing significantly. The scientific field of indoor localization and navigation has experienced rapid development through many studies that consider advanced mobile and communication technologies [1]. The purpose of developing new localization algorithms and navigation systems is to enable autonomous mobile systems to use these solutions in performing a specific task or to assist people who have lost their ability to navigate [2] (e.g., blind people, people with Alzheimer’s disease). A secure, user-friendly, and accurate localization method that can run on a smartphone could open the door to many innovative applications and create new businesses opportunities. The global indoor location market is expected to reach a worth of $40.99 billion by 2022 [3]. A low-cost indoor positioning system (IPS) is very helpful as it can guide people through airports, shopping malls, museums etc. [1,4]. In these environments, it can be very difficult to figure out where to go without spending a lot of time looking for directions. Accurate indoor localization represents a major challenge, mainly due to the fact that GNSS (Global Navigation Satellite System) signals are not available indoors [5]. The modern smartphone is increasingly used as a Personal Navigation System (PNS) because it contains many sensors (accelerometer, gyroscope, magnetometer, altimeter, camera) and communication modules (Bluetooth, WiFi, NFC, 5G, ultra-wideband), and, last but not least, almost everyone has it in their pocket [1,6]. Moreover, smartphones contain increasingly powerful multicore processors that allow the implementation of computationally intensive localization algorithms. A practical Indoor Positioning System (IPS) should have characteristics such as ease of implementation, acceptable localization accuracy, scalable system, feasible system cost and minimal computational complexity [7].

There are two main approaches to indoor localization, namely infrastructure-based and infrastructure-less approaches [8]. The latter generally uses fingerprints from environmental features such as sound, light, magnetic field or smartphone sensors. Infrastructure-based methods may use pre-installed visual sensors or wireless technologies such as ZigBee [9], WiFi, Ultra-Wideband (UWB) [3], Radio Frequency Identification (RFID) [10] and Bluetooth Low Energy (BLE) [1]. An infrastructure-based indoor positioning system can be expensive, either because of the methods required or because of the expensive hardware components. Among all these technologies, BLE is one of the most widely used in ubiquitous computing and many Internet-of-Things (IoT) applications because it has many advantages such as low power consumption and low cost [7,11]. BLE transmitters or beacons are portable and easy to deploy, they have the potential to provide high positioning accuracy, and they can provide advanced services to users. ZigBee consumes less energy than BLE, but it is not as widely supported on smartphones. WiFi is widely supported but it has a higher energy requirement than BLE [1]. Regarding Ultra-Wideband (UWB), which is an emerging technology (still not supported on smartphones) in the field of indoor positioning, Dardari et al. [12] provided a detailed comparative analysis of UWB positioning technologies.

Bluetooth low-energy transmitters are used in combination with various standards or protocols, such as iBeacon [13] and Eddystone [14], which define how data packets are transmitted. For localization purposes, the advertising mode, in which data packets are sent periodically, is most established. In this mode, beacon messages are advertised on the three primary channels 37, 38 and 39, to reduce interference with other wireless technologies (e.g., with WiFi channels 1, 6 and 11) and increase redundancy. In addition to broadcast transmissions, the advertising channels allow a device to be discovered and securely connected.

Radio-based indoor positioning can generally be divided into three groups: Proximity, Time of Flight (TOF) measurements, and Received Signal Strength Indicator (RSSI)-based methods [15]. In some cases, these have also been supplemented with an Angle of Arrival (AOA) localization approach [16]. Depending on the localization approach chosen to calculate the current position, the following methods are most commonly used: Triangulation, trilateration and fingerprinting. Among these, fingerprinting is perhaps the most popular due to its simplicity: It is based on signal strength (RSSI) and its procedure is basically to collect the signal from the transmitters and assign it to a specific position. It consists of two phases: The offline (the calibration or training) phase [11] and the online (the positioning) phase [17]. The most common fingerprinting matching algorithms can be classified as: (a) probabilistic; (b) deterministic, such as k-nearest neighbor (or weighted k-nearest neighbor); and (c) machine learning- and sparse sampling-based [18,19]. Positioning using BLE fingerprinting has the potential to achieve high accuracy if sufficiently dense training data are available. However, this process is time- and labor-intensive, which is its main drawback [20]. Another problem associated with the fingerprinting method is that the time complexity of the execution phase increases with the size of the localization area. Moreover, the instability of RSSI in the indoor environment forces frequent updating of the radio map database [21]. Therefore, a better alternative to the fingerprinting approach could be model-based methods, among which trilateration is the most popular [15]. Localization solutions based on trilateration mostly use path loss models to estimate the range from the RSSI of the available beacons [11]. In addition to the trilateration localization method, weighted centroid localization also relies on the signal propagation model to estimate the distance from the RSSI [22]. The advantages of using models over the fingerprinting approach are a more effortless adjustment of the radio maps as the environment changes and better extrapolation of RSSI signals for areas where measurements have not been made.

The main challenge in fingerprinting- and model-based IPS is that accuracy is affected by several factors, such as signal-related and environment-related factors [15]. In terms of signal-related factors, the following can often occur: Large fluctuations, reflection, path loss, non-line-of-sight conditions and multipath fading, and many of these factors also depend on the type of material present in the environment [23]. For environment-related factors, the main ones are changes in hardware or furniture, the presence of people, or ambient humidity conditions [24].

Regardless of the localization technology used, there are several more or less labor-intensive approaches to collect localization data in the modeling or fingerprinting calibration phase, which can be classified as follows [11,18,20]:

- A fully manual approach consisting of a calibration phase, e.g., the traditional manual survey, where the user collects the signal at discrete and uniformly distributed survey points.
- A semi-automated approach that attempts to reduce the time and effort of the calibration phase, e.g., with the use of interpolation-based methods, the user attempts to construct a signal map from a sparse set of fingerprints collected while walking through a space.
- A fully automated approach that does not require any calibration phase. It uses only online RSSI measurements or those that in some way merge the calibration phase and the positioning phase, e.g., implicit or explicit crowdsourcing that involves users in data collection.

An automated system has many advantages in various approaches to data collection, e.g., quick and easy construction of datasets for model development or fingerprinting, beacon parameter studies, in-depth studies of beacon location and density in a particular environment, and rapid maintenance of the database as the environment changes [8,20].

### Related Work

In this section, the works related to the study presented in this article are analyzed. First, an overview of the most commonly used technologies in indoor positioning is given, with a later focus on the technologies that use radio frequency signals, with a greater emphasis on BLE technology.

Self-localization of Unmanned Ground Vehicles (UGV) in indoor environments is already a well-developed field, as they can be equipped with more powerful hardware and additional sensors, e.g., depth camera, stereo camera, LIDAR, ultrasonic sensors, etc. [25]. In conjunction with the sensor LIDAR, the method of simultaneous localization and mapping (SLAM) [26] in particular has become established, which can also process the information obtained from the camera [27] (visual SLAM). Within the visual localization approach, several algorithms have been developed to determine an agent’s motion (person, vehicle, robot) to which the camera is attached. The established methods include: Simultaneous Localization and Mapping (SLAM) [27], Visual Odometry (VO) [28,29], Structure From Motion (SFM) and image-to-map matching [30]. The SLAM and SFM methods are fairly computationally demanding since they construct a 3D map of the environment in addition to the motion estimation.

For indoor localization purposes, the Inertial Navigation System (INS) [16,31] based on the Inertial Measurement Unit (IMU) is often used as a complementary system to radio- or visual-based localization. The pedestrian inertial navigation system, which uses a dead reckoning approach, usually consists of a pedometer (or step counter) and a digital compass, which enable to calculate the current pose according to the starting point [3,32].

According to the currently existing solutions and studies [33], the most suitable smartphone indoor localization approach is based on the measurement of WiFi [2], Bluetooth [34] and geomagnetic field signals [35]. However, localization methods based on only one technology or sensor often cannot provide the required positioning accuracy, so new approaches based on sensor fusion [3,36] are needed. Many studies show that a combination of inertial sensors and camera-based techniques has great potential since they provide better robustness and higher localization accuracy [37]. The fusion of data from a camera and inertial sensors can be performed using Kalman filters as proposed by Sirtkaya et al. [38]. Since image processing places a heavy load on the processor and therefore the battery, visual localization is not always the best choice for indoor localization. Using visual localization within a smartphone application can also be inconvenient as the user has to constantly provide the correct direction of the camera according to the environment [39]. This is not necessary with the radiolocation, which is a significant advantage.

The disadvantage of indoor radiolocation over visual localization is that it requires some infrastructure. From this point of view, WiFi localization takes precedence over Bluetooth-based localization because WiFi networks are already present in most buildings. However, WiFi localization does not enable high localization accuracy in existing networks since WiFi access points are usually rather sparsely located [2]. Due to this disadvantage in WiFi localization, Bluetooth Low Energy (BLE) [5,40] has been established for the purpose of indoor localization. BLE has many advantages over WiFi: from the low price of transmitters, low power consumption and better robustness to the smaller size of integrated circuits [41,42]. Li and Ma [43] took a hybrid approach using BLE and WiFi. They used BLE tags and BLE/WiFi repeaters. Their system performs position calculations based on two values: RSSI fingerprint and cell of origin. They conducted experiments in a rest area and an office area and obtained errors of 1.2 and 1.37 m for these areas, respectively.

In the field of radiolocation, three approaches based on an analysis of Bluetooth signal strengths have been established: Methods that consider the strongest base station; methods that require the construction of path loss models, i.e., models of signal strengths (and use trilateration) [1,11,24]; and methods based on the principle of “fingerprints” [21,42]. Tosi et al. [44] reviewed in their study the main methodologies adopted to investigate BLE performance: they provided an analysis of the maximum number of connectable sensors, throughput, latency, power consumption and maximum achievable range, with the aim of identifying the current limitations of BLE technology. Zue et al. [45] proposed a graph optimization-based approach that combines fingerprinting-based methods and range-based methods. The authors performed a test in an area of 90 m × 37 m with two different numbers of beacons, namely 24 beacons (sparse) and 48 beacons (dense), to see the effect of beacon density on the position estimates. They obtained errors of 2.26 and 1.27 m with the sparse beacon environment and dense beacon environment, respectively. In [46], the authors implemented a fingerprinting algorithm with fuzzy logic type-2 that is suitable for use as an indoor positioning method with BLE beacons with an average localization error of 0.43 m. Since this solution is based on fingerprinting, it requires a time-consuming offline phase and needs several additional algorithms for support. Thus, the computational resource consumption and algorithmic program complexity are comparatively high.

In previous studies, researchers have already focused on developing automated or semi-automated data acquisition systems [47]. Peng et al., developed an efficient method to create and update a fingerprint database using an Unmanned Ground Vehicle (UGV) platform. To collect BLE and WiFi fingerprints, they used a smartphone installed on the UGV. According to the obtained results, the root mean square error of the positioning results was reduced by 20% compared to the traditional fingerprint collection methods. Gao et al. [48] proposed the so-called path survey technique, in which radio maps are created from a sparse set of BLE and WiFi fingerprints. These were collected in such a way that a person walked through a room. With the obtained results, they showed that with a path-survey technique (and using Gaussian processes), the created maps are of the same quality as in the case of manual collection. The authors of [18] proposed a semi-automated fingerprint collection system (static rotating platform) to simplify and speed up the data collection process for 50%. Similarly, Nastac et al. [49] discussed the problem of automated data acquisition in fingerprint-based indoor positioning. As a platform for collecting data, they used a robot with simple wheel encoder sensors for odometry-based positioning. The benefit of using a robot was most evident in the time savings, as the data acquisition time could be reduced from 16 to 2 h for 3000 observations. In the process, the measurement accuracy could also be increased compared to the manual approach.

When setting up a BLE network, the main challenge is to mark the BLE transmitters’ locations on the map of the space and then collect the RSSI measurements for constructing the path loss model. These tasks are rather time-consuming since the network operator spends at least half a minute to measure the locations of each beacon (e.g., using a tape measure) or to collect the signal strengths at each measurement point. Therefore, this paper presents a fully automated approach for data collection and online model development on a smartphone, which is a significant advance over state-of-the-art solutions. The latter have one of the drawbacks, such as the requirement for manual measurement and inserting the beacon’s locations, offline data processing, or the use of expensive accessories, such as a wheeled robot. The proposed system allows real-time determination of in which part of the space there is a lack of measurements or which transmitter model is not yet good enough. With the implemented fusion of visual SLAM and inertial navigation on a smartphone, the proposed system enables accurate localization during BLE data collection, which is a prerequisite for good models of signal strengths. These are used for parallel online localization with the Particle Swarm Optimization (PSO)-based method, which enables reinitialization of the SLAM algorithm and real-time validation of the models. In the online model development phase, the beacon’s locations and the model parameters are calculated using the constrained nonlinear optimization method. Assume that the model construction completion criterion is met and the BLE localization accuracy is satisfactory. In this case, the visual SLAM algorithm can be turned off and the position is calculated using only the PSO method, which results in a lower CPU and battery load. In contrast to fingerprinting methods, the proposed system is quickly adaptable to changes in the room (e.g., a new beacon or furniture layout) and supports a crowdsourcing approach to data collection.

The paper is organized as follows. Section 2 describes a fully automated data collection approach based on visual-inertial SLAM. Section 3 provides the background to the online path loss model construction on a smartphone. In Section 4 the particle swarm optimization (PSO) method is proposed and described in detail. Section 5 presents the results of path loss model construction and Section 6 deals with pedestrian localization using the proposed algorithm. A discussion and concluding remarks are presented in Section 7 and Section 8, respectively.

## 2. SLAM-Based Fully Automated Approach for Data Collection

**Figure 1:**
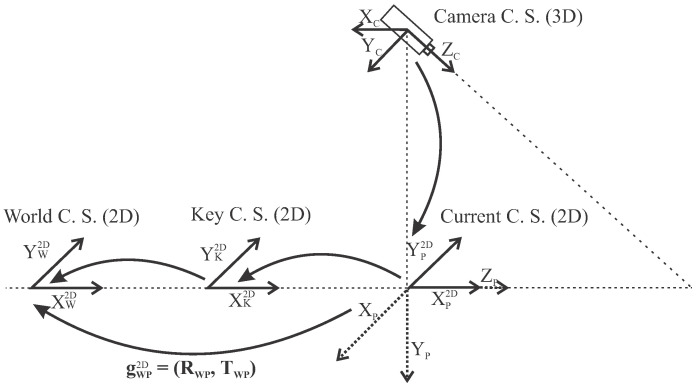
The ARCore tracking algorithm calculates transformation from camera C. S. to world C. S.

When gathering measurements of BLE signal strengths, real-time localization that enables accurate determination of reference positions in space is essential. In previous studies, we have already shown that an algorithm combining an inertial and visual navigation system is best suited for this purpose [37,50]. We have developed a visual odometry that can very accurately determine the position of the smartphone in the short term by tracking incremental movements. The problem with visual odometry is that the position drifts over time and the error accumulates [51]. To improve the localization accuracy over time using a camera and the smartphone’s inertial sensors, we used Google’s ARCore library [52] in our fully automated system for data acquisition and model development. The ARCore algorithm is based on the state-of-the-art visual-inertial SLAM [53,54] or Concurrent Odometry and Mapping (COM) [55], which allows determining the pose of a smartphone with a monocular camera in an unknown environment, such as ORB-SLAM [56], MIMC-VINS [57] or LSD-SLAM [27]. Feature detection helps in calculating the relative change in position by searching for the same unique feature between frames. Using motion tracking capabilities, ARCore tracks the smartphone’s position relative to the world Coordinate System (C. S.) by identifying key feature points (see Figure 1). This visual information is combined with inertial measurements (i.e., linear acceleration and angular velocity) from IMU to estimate the relative 6-DOF pose (position and orientation) of the smartphone with respect to the world coordinate system. ARCore has the ability to track up to 20 images simultaneously, with tracking occurring on the smartphone. In addition, ARCore can store up to 1000 reference images in an image database.

ARCore’s tracking algorithm computes the 3D transformation from the world C. S. to the camera C. S. (Figure 1). To simplify pose representation, a 3D-to-2D transformation is required. First, to obtain the current 2D pose of a smartphone in the world C. S., the camera C. S. must be transformed to the current C. S. (from 3D to 2D), and second, the transformation $g_{\mathrm{WP}}^{2D}$ from the current C. S. to the world C. S. must be determined. The rotation matrix $R_{\mathrm{WP}}$ determines the heading and the translation vector $T_{\mathrm{WP}}$ determines the position of the smartphone in 2D space [37]. The world C. S., which is determined by the first frame, is not movable and it represents a reference C. S. in which the final result of the visual localization is expressed.

**Figure 2:**
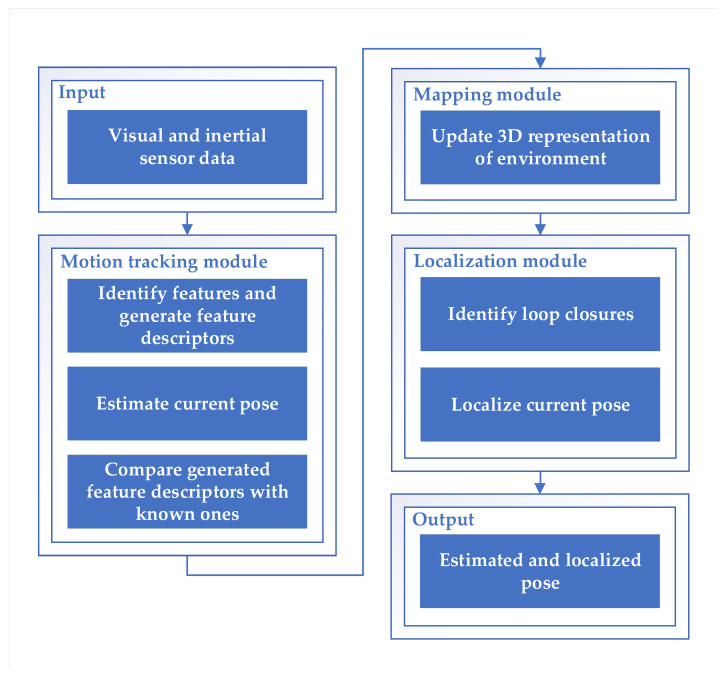
The concept of the ARCore concurrent odometry and mapping algorithm [55].

The concept of the ARCore COM algorithm is shown in Figure 2. The algorithm is divided into three main modules:

- Motion tracking module;
- Mapping module;
- Localization module.

ARCore takes visual data (captured images from smartphone camera) and inertial data (from IMU) as the main input sources. Each of the modules has a clear responsibility for the continuous COM process and they are highly dependent on each other.

**The motion tracking module**, also known as the front-end module, receives input data to compute a locally accurate pose estimate. From the visual data, it identifies good feature points and provides the captured feature descriptors. These descriptors also define orientation, gravity direction, scale and other aspects. The estimated pose is based on the feature descriptor correspondences from the previous frame. This module maintains only a limited history of tracked motion and treats any previously generated estimated feature point pose as fixed. Inertial sensors help correlate spatial features observed in one frame with spatial features observed in a subsequent frame to efficiently determine the change in pose. Therefore, the estimated pose can be performed with high frequency (due to the limited matching time) with a locally accurate pose. On the other hand, the estimated pose has almost no control over the drift or no means to check for loop closure since only a very limited history is stored.

**The mapping module**, also called the back-end module, takes as input the previously defined feature descriptors from the current frame. In this module, a 3D representation of the environment is created based on a stored variety of maps, feature descriptors, and estimated device location. It has an extensive history of 3D feature positions in the environment and poses of the smartphone. Matching the newly generated features with the fixed features in the multitude of maps is an extensive process. Therefore, the updating is done with a relatively low frequency. The 3D representation is sent to the localization module. Within the mapping module, ARCore provides spatial understanding based on plane detection in the depth map. Using the same feature points used for motion tracking, ARCore looks for clusters of points that appear to lie on common surfaces. These can be anything, such as floors, tables, or walls. These surfaces are stored as planes with a specific boundary.

**The localization module** identifies discrepancies between stored and observed feature descriptors. It performs loop closure by minimizing these discrepancies (e.g., with a least squares adjustment or bundle adjustment [58]) to output a localized pose. If the error (difference between estimated pose and matched pose) is greater than a certain threshold, the localized pose snaps to the pose from the descriptor matching. This can happen for a variety of reasons (e.g., significant drift). Difficulties occur for real-world surfaces without texture (e.g., white wall) because ARCore uses feature points to detect surfaces. No useful features can be found in textureless regions. In case the SLAM algorithm fails, inertial navigation based on a pedometer and a digital compass becomes crucial. Inertial navigation is combined with visual localization using an extended Kalman filter (as described in our previous work [37]).

In summary, the ARCore algorithm performs two types of outputs from the input data (see Figure 2). First, it provides a locally accurate and high frequency estimated pose with no control segment such as loop closure or drift correction. Second, it provides a localized pose with a lower update frequency due to extensive steps such as descriptor matching (with the full history) or an adaptation process.

Suppose path loss models for the current space do not already exist on the server. In that case, the visual-inertial localization method described above allows any smartphone application user to begin collecting BLE signal strength data for online path loss model construction and subsequent radiolocation. The smartphone user only needs to walk through the room while the visual-inertial localization system simultaneously determines their current pose and creates a visual and radio map of the environment. While using the smartphone application, it collects the following data into the database: BLE signal strengths with time and pose (position and orientation of the smartphone) where individual RSSI of a beacon with a unique ID is captured. The local data stored on the smartphone are used for online model construction, as described in the next section. The collected data, along with the path loss model parameters, are also sent to the server, where they are used for further post-processing and realization of the crowdsourcing approach.

## 3. Online Path Loss Model Construction

Measurements of BLE signal strengths can be described using a path loss (or path attenuation) model that determines a reduction in signal strength as the receiver moves away from the transmitter. The model has the general form of a nonlinear equation with three parameters:

(1) $$d=K_{1}\cdot ratio^{K_{2}}+K_{3},$$

where ratio=R/TXP=R/−59 (TXP=−59 dBm represents a signal strength at distance of d=1 m). From Equation (1) the signal strength *R* can be expressed:

(2) $$R=TXP\cdot {\left(\frac{d-K_{3}}{K_{1}}\right)}^{K_{2}^{-1}},$$

where the distance *d* is equal to:

(3) $$d=\sqrt{{\left(x-x_{0}\right)}^{2}+{\left(y-y_{0}\right)}^{2}+h^{2}}.$$

Equation (3) contains the position (x,y) at which the signal strength *R* is measured, the location of the beacon in space $\left(x_{0},y_{0}\right)$ and the distance *h*, which represents the height difference between the smartphone and the known height at which the beacon is fixed. Constrained nonlinear optimization (in which a trust-region method [59,60] is used) can be used to determine the model parameters $K_{1}$, $K_{2}$, $K_{3}$ and the position of the beacon in space $\left(x_{0},y_{0}\right)$ in a way that model (2) fits the measurements well. Since the optimization problem has multiple solutions, it is very important to strictly set the boundaries of model parameters and limit the space of beacons’ locations.

### Constrained Nonlinear Optimization

Constrained nonlinear minimization [61,62,63] involves finding the vector *x*, representing the local minimum of the scalar function f(x), taking into account the given constraints on the vector *x*. These constraints can be given as: A linear constraint (A·x≤b,Aeq·x=beq), a nonlinear constraint (c(x)≤0orceq(x)=0) or a constraint with given bounds (box constraints) l≤x≤u. In the following, the emphasis will be on nonlinear optimization with box constraints *l* (vector of lower bounds) and *u* (vector of upper bounds). In this case, we solve the following problem:

(4) min{f(x)suchthatl≤x≤u}.

In the vector of lower and upper bounds, some of the components may be unconstrained (equal to ±∞). In the optimization process, the method returns a sequence of strictly feasible points. With the goal of preserving feasibility while achieving robust convergence behavior, the following techniques are used. In the first technique, the unconstrained Newton step is substituted with a scaled modified Newton step. In this way, the two-dimensional subspace *S* is defined using the preconditioned conjugate gradient method [64]. The latter ensures global convergence. In the second technique, reflections [61] are used to increase the step size. The scaled modified Newton step is obtained by considering the Kuhn–Tucker necessary conditions for Equation (4):

(5) $${\left(D\left(x\right)\right)}^{-2}g=0,$$

where D(x) is a diagonal scaling matrix:

(6) $$D\left(x\right)=diag\left({\left|r_{k}\right|}^{-1/2}\right).$$

The vector r(x) is defined below according to the gradient g=∇f(x) and bounds, for each 1≤i≤n:

- If $u_{i}<\infty$ and $g_{i}<0$ then $r_{i}=x_{i}-u_{i}$;
- If $l_{i}>-\infty$ and $g_{i}⩾0$ then $r_{i}=x_{i}-l_{i}$;
- If $u_{i}=\infty$ and $g_{i}<0$ then $r_{i}=-1$;
- If $l_{i}=-\infty$ and $g_{i}⩾0$ then $r_{i}=1$.

It is important to point out that the nonlinear system in Equation (5) is not differentiable when $r_{i}=0$. However, by taking into account the restriction l<x<u and maintaining strict feasibility, such points can be avoided. According to the nonlinear system of Equation (5), the scaled modified Newton step $s_{k}$ is computed as the solution of the linear system:

(7) $${\hat{M}}_{k}D_{k}s_{k}^{N}=-{\hat{g}}_{k},$$

at the *k*th iteration, where:

(8) $${\hat{g}}_{k}=D_{k}^{-1}g_{k}=\mathrm{diag}\left({\left|r_{k}\right|}^{1/2}\right)g_{k}$$

and:

(9) $${\hat{M}}_{k}=D_{k}^{-1}H_{k}D_{k}^{-1}+\mathrm{diag}\left(g_{k}\right)J_{k}^{r}.$$

In Equation (9), $J^{r}\left(x\right)$ represents the Jacobian matrix of $\left|r(x)\right|$ and *H* is the Hessian matrix. The Jacobian matrix $J^{r}$ is diagonal, where each element is equal to 0, −1 or 1. For the case where all elements of *l* and *u* are finite, the Jacobian matrix is equal to $J^{r}=\mathrm{diag}(\mathrm{sign}\left(g\right))$. If there is a point at which $g_{i}=0$, then $r_{i}$ might not be differentiable. However, at such a point the Jacobian $J_{ii}^{r}=0$ is determined. Such non-differentiability is not a problem, since it is not important which value $r_{i}$ takes. Indeed, the function $\left|r_{i}\right|\cdot g_{i}$ is continuous, although $\left|r_{i}\right|$ is discontinuous at this point.

Equation (7) considers the use of the affine transformation $\hat{x}=D_{k}x$ (the matrix $D_{k}$ is a symmetric matrix: $D_{k}=D_{k}^{T}$). This transformation converts the constrained nonlinear optimization problem into an unconstrained nonlinear optimization problem, minimizing the function in the new $\hat{x}$ coordinates. This means that in this case the following minimization problem is solved:

(10) $$min\{{\hat{\psi }}_{k}\left(\hat{s}\right)=\frac{1}{2}{\hat{s}}^{T}{\hat{M}}_{k}\hat{s}+{\hat{g}}_{k}^{T}\hat{s}\;\mathrm{such}\;\mathrm{that}\;∥\hat{s}∥\leq \Delta_{k}\}$$

or, if $s=D_{k}^{-1}\hat{s}$ is considered, then the following minimization problem can be solved in the original space:

(11) $$min\{\psi_{k}\left(s\right)=\frac{1}{2}s^{T}M_{k}s+s^{T}g_{k}\;\mathrm{such}\;\mathrm{that}\;∥D_{k}s∥\leq \Delta_{k}\},$$

where:

(12) $$M_{k}=H_{k}+C_{k}$$

and:

(13) $$C_{k}=D_{k}\mathrm{diag}\left(g_{k}\right)J_{k}^{r}D_{k}.$$

In Equation (10) $\Delta_{k}$ is a positive scalar representing confidence interval width. During the optimization, it is adjusted according to the rules of the trust-region method [59,60].

The second technique of the constrained nonlinear optimization provides feasibility with the following condition l≤x≤u to be satisfied. Within this technique, so-called reflections are used, which have the purpose of increasing the step size. A single reflection step can be described as follows. If *p* represents a step that exceeds the first bound constraint (the *i*th upper or *i*th lower bound), then the reflection step is defined as $p^{R}=p$ and $p_{i}^{R}=-p_{i}$ for the *i*th element for which a constraint is required.

## 4. Localization with Particle Swarm Optimization

Particle Swarm Optimization (PSO) is an optimization method that was initially described by Kennedy, Eberhart and Shi [65,66]. In a practical application [67], they showed that the PSO method could be used to simulate social behavior within a school of fish or a flock of birds. PSO is a stochastic optimization method that robustly solves a given problem in successive iterations according to the selected objective function. This determines whether the candidate solutions of the problem are appropriately improved at each step. The candidate solutions are represented by particles forming a particle swarm. The solution to the optimization problem can be achieved by moving these particles within a constrained region using simple mathematical formulas. These determine a new position and velocity of the particles in each iteration. In the case where all particles converge to a common position, the global optimum of the objective function is found.

Let the position of the particle be denoted by $x_{i}\in R^{n}$ and its velocity by $v_{i}\in R$. In the swarm with *S* elements, the best position of each particle is denoted by $p_{i}$ and the best position of the whole swarm is denoted by g. The number of all required particles *S* can be inferred from the experimental results or calculated using the suggested formulas (e.g., $S=2\sqrt{n}+10$, where *n* is the search area’s dimension).

In the PSO method [66] a new position of particle $x_{i}^{k+1}$ is determined by the model of particle movement that takes into account its current current velocity $v_{i}^{k}$, position $x_{i}^{k}$, the distance $d_{p_{i}}^{k}$ between the particle’s best known position $p_{i}^{k}$ and the particle’s current position $x_{i}^{k}$ and the distance $d_{g_{i}}^{k}$ between the best position of the whole swarm $g^{k}$ at a given time and the particle’s current position $x_{i}^{k}$:

(14) $$x_{i}^{k+1}=x_{i}^{k}+v_{i}^{k+1},$$

where:

(15) $$v_{i}^{k+1}=\omega v_{i}^{k}+\Delta v_{i}^{k},$$

(16) $$\Delta v_{i}^{k}=\varphi_{g}r_{g}\left(g^{k}-x_{i}^{k}\right)+\varphi_{p}r_{p}\left(p_{i}^{k}-x_{i}^{k}\right)=\varphi_{g}r_{g}d_{g_{i}}^{k}+\varphi_{p}r_{p}d_{p_{i}}^{k}.$$

The parameter ω can be used to determine whether accurate local exploration (a low value) or faster convergence (a high value) is preferred. The parameter represents the remembering rate or inertia weight [66], which determines to what extent the previous direction of the particle is preserved. The parameter $\varphi_{g}$ is a swarm confidence factor (or a social learning factor) and the parameter $\varphi_{p}$ is a self confidence factor (or cognitive learning factor). The parameters $r_{g}$ and $r_{p}$, which change randomly in the interval [0,1], represent random accelerations in the directions of the best position of the particle $p_{i}$ and the best position of the whole swarm g. In the process of the PSO configuration, according to a given optimization problem, great emphasis should be put on the choice of parameters ω, $\varphi_{p}$ and $\varphi_{g}$, which have the most significant impact on the performance of the method [68,69,70,71].

The goal of PSO-based localization is to find a particle (i.e., the position of the smartphone) (x,y) in two-dimensional space for which the corresponding vector of signal strengths $R_{P}=\left[f_{R_{1}}\left(d_{1}\right),f_{R_{2}}\left(d_{2}\right),\ldots ,f_{R_{m}}\left(d_{m}\right)\right]$, obtained by the constructed path loss models $R_{i}=f_{R_{i}}\left(d_{i}\right)$ (for i=1,…,m), is the most similar to the vector of current measurements of signal strengths $R_{M}$ according to the objective function:

(17) $$f_{c}=\sum_{i=1}^{m}\left|R_{M_{i}}-R_{P_{i}}\right|,$$

where *m* is the number of BLE transmitters considered for the calculation of the current position. The distance $d_{i}$ (for i=1,…,m) represents the Euclidean distance between the current position of the smartphone (x,y) and the position of the *i*th BLE transmitter $\left(x_{0_{i}},y_{0_{i}}\right)$.

Using the path loss models, the PSO localization method generates fingerprints for all particles in the swarm that represent the local radio map. The particles are uniformly distributed in the grid of the chosen size at the beginning of each optimization.

## 5. Online Path Loss Model Construction Results

**Figure 3:**
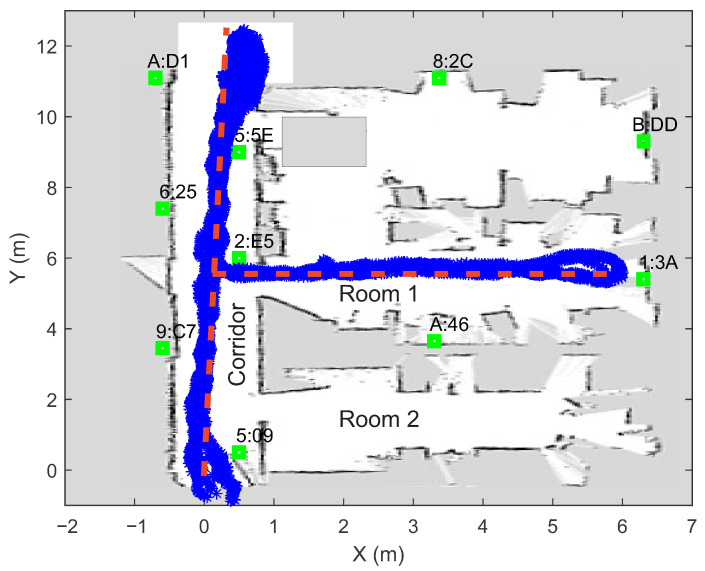
Locations of BLE beacons (green squares) and walked path (determined with the visual-inertial SLAM) during automated measurement capture. The ground truth path is marked with the red dashed line. The white pixels represent empty space and grey pixels represent occupied space.

In order to evaluate the proposed system for automated localization data collection, online modeling and real-time PSO localization on a smartphone (Samsung Galaxy S9), an experiment was performed in am ∼80 m^2^ laboratory, where ten Bluetooth beacons from Kontakt.io [72] were installed. The beacons were distributed around the room (taking into account certain constraints due to the layout of walls, doors, pillars and windows), as shown in Figure 3 (green squares). To reduce the influence of the presence of human bodies on the measurements of the signal strengths, the transmitters were mounted at the height of 2 m above the floor.

The measurements of the signal strengths from all ten beacons were collected in a way that a pedestrian repeatedly walked the path that is shown in Figure 3 while the smartphone recorded the data. The current position of the smartphone (calculated every 50 ms) was tracked by the algorithm combining the SLAM and the inertial navigation system (see Section 2). All of the positions where the measurements of the signal strengths were performed can be seen in Figure 3. This figure shows that the obtained position points deviate slightly from the intended route (the lines were marked on the ground). Part of this error is due to the visual-inertial localization algorithm (strong magnetic interference affects the digital compass) determining the position relative to the starting point, and part is due to the inaccurate walking of pedestrians along the line. Our results on visual localization include that under optimal environmental conditions, the errors are at most of a few tens of centimeters in a typical room-sized environment (without significant drift errors). However, as the speed of movement increases and in low light conditions, the overall performance may decrease.

**Figure 4:**
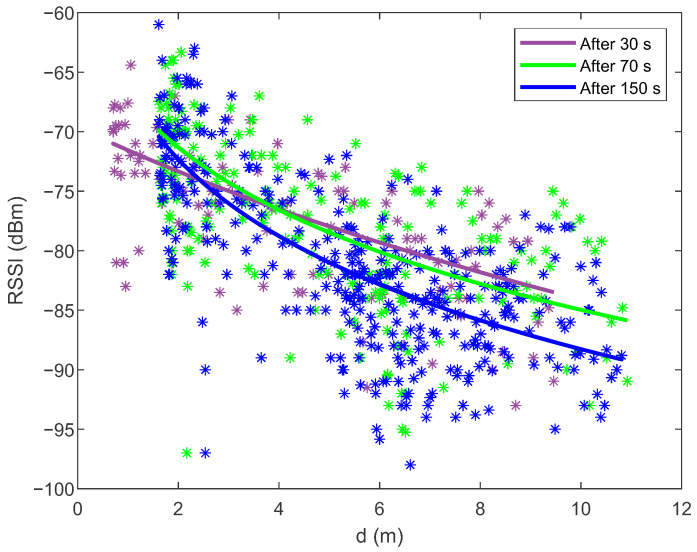
Fitting the online constructed path loss models (2) to the measurements of the signal strengths after 30, 70 and 150 s of walking (for the BLE beacon with MAC address DA:57:30:EC:6A:D1). Since the data contain a lot of noise, the coefficient of determination for all models is quite low: $R^{2}=0.5$.

Using the constrained nonlinear optimization and the signal strengths measurements from all Bluetooth beacons distributed in the room, the path loss models are constructed online on the smartphone. Figure 4 shows the fitting of the models to the measurements of the signal strengths after 30, 70 and 150 s of walking (for the BLE beacon with MAC address DA:57:30:EC:6A:D1 and position (−0.7,11.10)) to demonstrate the influence of the amount of the collected measurements. After 30 s of walking, the pedestrian covered a distance of about 10 m along the corridor. After 70 s, half a *T*-shaped path was covered, and after 150 s, the pedestrian covered the entire *T*-shaped path (i.e., 36 m) from the start and back. The online construction of the path loss models and the determination of the beacons’ locations are performed in iterations every 10 s when enough new data are collected. The constrained nonlinear optimization runs in parallel to the real-time PSO localization on the smartphone, which means that they do not affect each other when individual CPU cores are fully utilized.

In the laboratory where the experiment was conducted, there were many obstacles (walls, wooden barriers, pillars) between the transmitters and the receiver (smartphone), while the pedestrian walked along the path shown in Figure 3. Due to absorption and reflection from the obstacles, the signal strength measurements were very noisy and scattered at the same distance from the beacon (see Figure 4).

**Figure 5:**
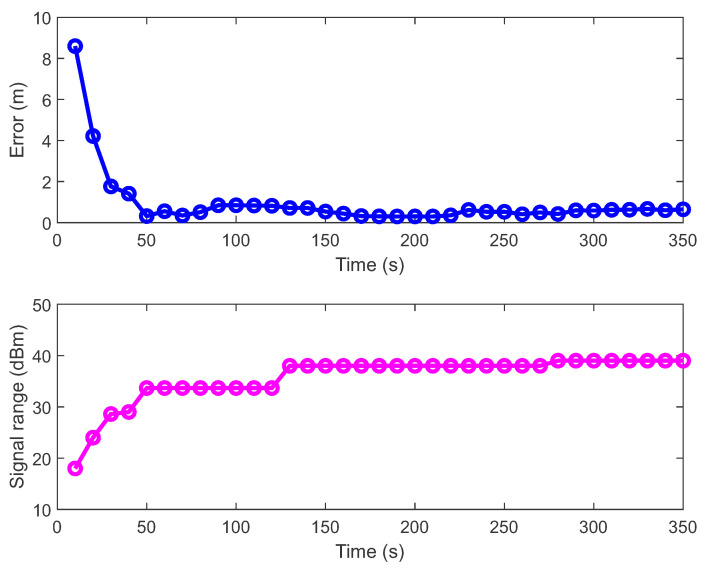
The upper part of the figure shows the error between the beacon’s actual location (with MAC address DA:57:30:EC:6A:D1) and the position calculated by the constrained nonlinear optimization method. The error depends on the time or the travelled distance. The lower part of the figure shows the BLE signal range (the difference between maximum and minimum signal strength) as a function of time or the travelled distance.

During data collection and online model development, the measurements and the path loss models shift left and right according to the distance *d* (see Figure 4) since the positions of the beacons are estimated with the constrained nonlinear optimization algorithm in addition to the model parameters. With more available data, the positions of the beacons converge towards the correct position (see the upper part of Figure 5). Still, after a certain number of iterations, they do not improve any more because the measurements contain a lot of noise. For model improvement, it is crucial to collect measurements at different distances (from 1 m to 10 m) from the beacon because, in this way, the whole RSSI signal range is covered (e.g., from −59 to −100 dBm). The lower part of Figure 5 shows the BLE signal range (the difference between maximum and minimum signal strength) as a function of the travelled distance. As the signal range increased, the error of the beacon’s location decreased. When the signal range reached values close to 40 dBm, the error entered the steady-state. After 30 s of data acquisition, the amount of data was still too small or the measurements are not well distributed throughout the room, which is reflected in the transmitter’s inaccurate position and a worse model 1 in Figure 4. After 70 s, the signal range and the beacon’s location were satisfactory and the path loss model was also close to the optimum. With the additional data, the models and beacons’ locations can be improved only slightly.

In the online data collection process and model development, it is essential to know when there is enough data for a given beacon. For this purpose, two (stopping) criteria can be used, first, when the beacon’s location changes less than the selected threshold during two consecutive iterations (in our case 0.1 m) and second, when the signal range is large enough (in our case 35 dBm). The duration of collecting the measurements and the path length are worse for estimating whether there are enough measurements. Namely, if the measurements are collected only in the same position (for a long time) or at a long distance from the transmitter, they are not useful for the model construction.

Once the positions of all beacons and the path loss models are computed, the goal of the proposed system is achieved. However, if there are any changes to the space (e.g., change in the position of the beacons), the whole process can be repeated very quickly. The data and models can be created by one user or by multiple users of the smartphone application. With the crowdsourcing approach, large amounts of data can be collected on the server, which means that the models created online can definitely be improved offline using more sophisticated and computationally intensive methods (e.g., using the SUHICLUST algorithm that creates fuzzy path loss models with confidence intervals [50]), if necessary.

## 6. Particle Swarm Localization Results

In order to evaluate the online developed path loss models and the PSO-based localization algorithm, the measurements of signal strengths were collected with a smartphone along the same path used to construct the models and determine the beacons’ locations (see Figure 3). As the pedestrian walked along the path, each time the smartphone receiver measured at least one new signal strength that was greater than −95 dBm, a vector of signal strengths $R_{M}$ was created from which the current position was determined. In our experiment, this happened on average every 30 ms, with each beacon sending a packet every 100 ms. For each beacon, the measurements of the signal strengths were independently filtered using the Savitzky–Golay filter (with frame length 99) [73] to reduce variability. Each vector of current measurements (or fingerprint) consists of at least three signal strengths from different beacons. In our experiment, the fingerprints contained five elements, as this is optimal according to our previous study [50]. To determine the accuracy of the PSO localization algorithm in real time, the visual-inertial SLAM algorithm was used to record the positions where the vectors $R_{M}$ were created.

**Figure 6:**
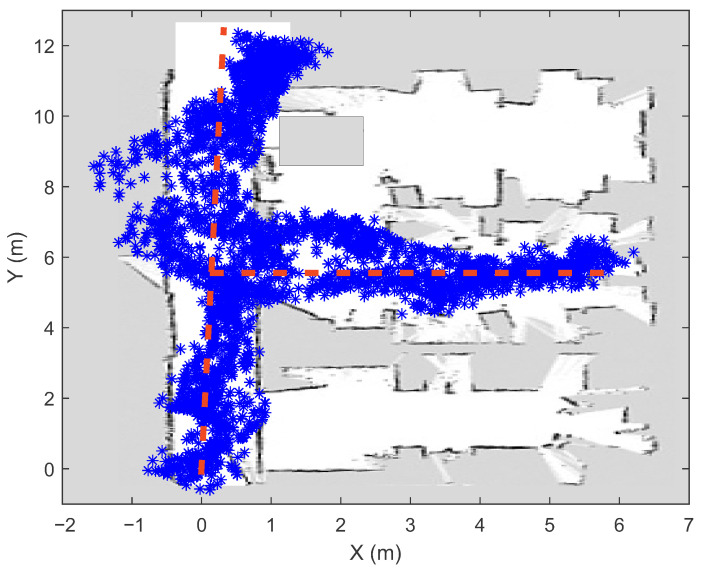
The indoor positioning results (after 5 min of walking) obtained using the online constructed path loss models and the PSO localization method.

During the experiment, the pedestrian walked for 5 minutes in the laboratory. Their current location (see Figure 6) was determined in real time using the PSO localization method on a smartphone (Samsung Galaxy S9), where the latest path loss models were used. These were created online from the latest available data and they were further improved with new data if the stopping criteria were not yet met (see previous subsection).

When selecting the PSO search area, the current position of the receiver (i.e., the smartphone) must be approximately known. During PSO initialization, the position can be calculated using the location of the nearest beacon (with the highest signal strength) or using the fast linear trilateration method. In this case, the room-level accuracy is acceptable. Thereafter, when PSO provides the first positioning results, the current position of the receiver can be used in the next iteration to determine the PSO search area (all particles are arranged around the current position).

With the aim of obtaining the optimal parameters of the PSO method, the offline simulation experiments were performed in the Matlab environment. In this way, the following parameters were obtained: ω=0.06, $\varphi_{p}=0.1$ and $\varphi_{g}=0.1$. The optimal size of the search area with a square shape is 0.8 m × 0.8 m. By choosing the boundaries of the search area, a filtering effect is achieved in addition to reducing the computational complexity.

Since the PSO algorithm must operate in real-time on a smartphone, the parameters were chosen as a trade-off between computational speed and localization accuracy. By running simulations, the number of particles and iterations needed was also determined. The use of eight particles and twenty iterations within the PSO optimization proved to be optimal, as a larger number of particles or iterations only increases the computational complexity and does not improve the result.

**Figure 7:**
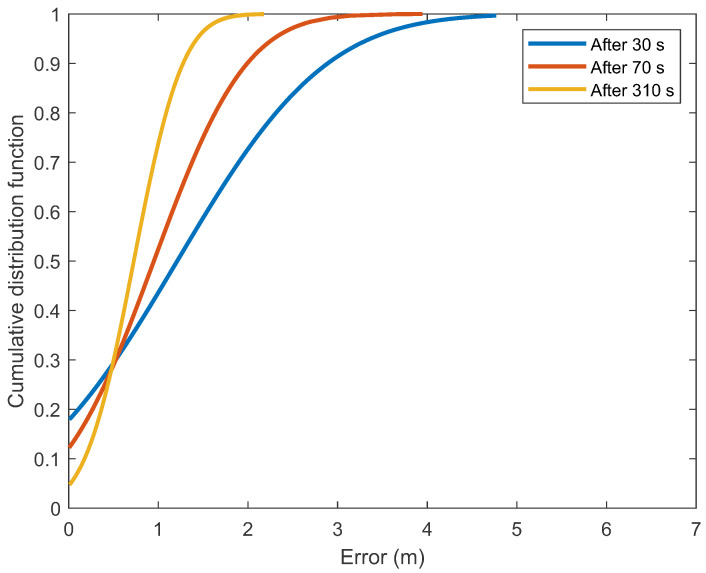
The cumulative distribution functions for the positioning errors in determining the positions by the PSO method after 30, 70 and 310 s of walking.

As path loss models and beacons’ locations were more accurately determined with more available data, the localization error decreased. The graph of the cumulative distribution function (Figure 7) shows that after 310 s of walking in 70% of the position estimates, the error was less than 1 m and the average error was less than 0.8 m. For comparison, when incomplete models built with a smaller dataset were used, the results show that after 30 s of walking in 44% of the position estimates the error was smaller than 1 m and after 70 s there were 52% of the position estimates where the error was smaller than 1 m.

**Figure 8:**
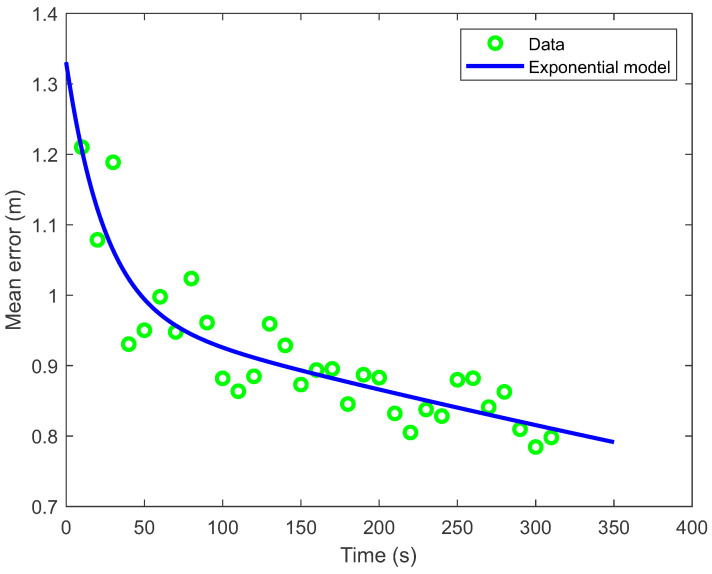
The mean localization error as a function of time or the travelled distance. The decrease of error can be described by an exponential model e=0.35exp(−0.04*t*)+0.98exp(−0.0006*t*) with the coefficient of determination $R^{2}=0.8$.

The results of the online construction of the path loss model show that in the first 80 s after data collection began, the models and the beacons’ locations changed drastically. Consequently, the positioning errors obtained with the PSO localization method (see Figure 8) were substantially larger in the first 80 s (or for the first eight model series). Thereafter, the mean positioning error decreased more slowly. The decrease of error can be described by an exponential model e=0.35exp(−0.04t)+0.98exp(−0.0006t) (where *t* is time) with the coefficient of determination $R^{2}=0.8$. In the first evaluation of the mean error, about 150 positions and in the last evaluation, about 5500 positions were used for error estimation. The number of calculated positions (with PSO method) used for estimating the mean error increased practically linearly with time. Since path loss model construction and PSO localization can run simultaneously on a smartphone, the comparison of the current radio and visual-inertial localization results can be used as a real-time criterion to determine in which part of space additional signal strength data need to be collected to improve the latest online computed models and consequently the localization accuracy.

When comparing the computational complexity, the localization algorithm based on the PSO method turns out to be better than the one based on the nonlinear trilateration [74], as it is 20 times faster for the same localization accuracy (on the smartphone, it spends about 5 ms computing a position). The experiment was performed on a Samsung Galaxy S9 smartphone (with a Samsung Exynos 9810 processor) using offline signal strength measurements and prebuilt path loss models. Average execution time for both localization methods was calculated from 5000 repetitions of the different position calculations.

## 7. Discussion

With the implementation of an automated system for BLE data collection, online path loss model construction, and real-time PSO localization on a smartphone, radiolocation becomes significantly less time- and labor-intensive by reducing configuration time (i.e., the time from the start of data collection to the moment when Bluetooth localization becomes operational) by more than 90%. With the manual collecting of signal strength data in the ∼80 m^2^ laboratory, where at least 80 measurement points are needed (previous studies showed that at least one measurement point per square meter is required), the network operator would spend about 40 min (half a minute to manually measure each location in the room and collect the signal strengths). To measure ten beacons’ locations in our laboratory, the network operator would spend an additional 5 min. A non-automated localization system also requires some time to transfer the data to the computer and compute the path loss models or create the map of fingerprints. For comparison, the proposed automated system required 5 min for collecting all signal strength data (more than 5000 measurement points), calculating path loss models, and determining all beacons’ locations.

Compared to our previous study [37,50,74], in which a visual odometry algorithm was developed to determine the reference positions during the collection of signal strengths, the new proposed automated system has many advantages. The visual-inertial SLAM does not require an initial camera calibration with a checkerboard to obtain intrinsic and extrinsic parameters describing the transformation between the camera and base coordinate systems. Therefore, the smartphone does not need to be at the same height above the ground during the walk. The visual-inertial SLAM also includes loop closure, which is very important to reduce position drift and error accumulation. Therefore, the proposed system can be used for data collection in wide area facilities such as airports, hospitals, museums, etc. The visual localization system based on SLAM is very accurate, but it can also fail, especially in dark places and places where the surfaces are without texture. In such a case, the fusion with inertial sensors and methods becomes crucial. The visual-based localization system has proven to be less suitable to be used as a primary localization system, as the image processing puts a heavy load on the processor, which drains the battery quickly. During the experiment, the two big cores of the Samsung Exynos 9810 processor (with four big cores operating at 2.9 GHz and four small cores operating at 1.9 GHz) were loaded more than 90% on average while using visual localization. By contrast, PSO localization is computationally much less demanding and can run on a small core (loaded less than 60% on average), saving energy.

In addition to the visual-inertial SLAM, another important component of the proposed system is the constrained nonlinear optimization, which allows constructing path loss models and determining the beacons’ locations online on a smartphone. The results of the experiment show that the beacons’ locations can be determined fairly accurately, as the final average errors are less than half a meter for all the beacons. These errors are not problematic since the obtained beacons’ locations can also be represented as virtual beacons’ locations that best fit the corresponding path loss models with the constrained parameters. In this way the deviations (due to the obstacles) in the path loss model can be partially compensated for with the virtual beacon’s location and consequently the model can better fit the measurements. It has been found that the constraints on the parameters of the path loss model can be quite stringent, since the same beacons located in open space have very similar path loss models. The constraints on the beacons’ locations do not impose an additional burden since the dimensions of the area where the transmitters are located are usually known. When implementing algorithms on a smartphone, the main concern is that they can run in real time or in limited time. Therefore, a trade-off between the accuracy and speed of the algorithms is required. Since the proposed system is designed to support the crowdsourcing approach, all the collected data are transferred to the server database where the models already created online can be additionally improved if needed. The proposed solution is suitable for both BLE network operators and end users. Anyone who enables visual localization on a smartphone can start simultaneous localization and data collection. Based on the proposed criteria (signal range, beacons’ location stability and accuracy of radiolocation compared to visual-inertial localization), the following information is available to the user collecting data: For which beacon in the room there are not enough measurements yet or in which part of the room the radiolocation is still poor and additional measurements are needed.

For real-time radiolocation on a smartphone, the PSO-based method was chosen instead of nonlinear trilateration. The reason is that PSO localization has been shown to be much faster. However, in order to achieve good localization accuracy, the parameters of the PSO optimization method must be carefully chosen, otherwise the algorithm may become slow and inefficient. The PSO localization is more flexible in the sense of a small temporary radio map generation, which consumes very little memory for the same accuracy as the fingerprinting method. The obtained localization results are slightly worse (with an average error of 0.8 m) than in the case where the locations of the beacons are known and the path loss models are constructed offline using more sophisticated methods [50] (with an average error of 0.5 m). Since most location applications (for large facilities such as airports) do not require high localization accuracy, the obtained results represent a good compromise according to the complexity of the network deployment and the localization accuracy.

The proposed system is not limited to collecting BLE signal strengths and PSO radiolocation. Depending on the needs, it can be modified to collect data about the WiFi, UWB or geomagnetic field signals and perform localization based on these signals.

## 8. Conclusions

This paper presents a sophisticated indoor localization system that combines visual-inertial positioning for accurate data collection and Bluetooth positioning for real-time smartphone tracking with low power consumption. The visual-inertial SLAM algorithm, which is part of the ARCore library, successfully fuses information from the camera and inertial sensors to provide accurate localization in large spaces over a long period of time. In this way, a suitable collection of measurements of signal strengths can be made quickly and easily, clearly defining which part of the space is not yet well covered by measurements. The path loss models and the beacons’ locations are determined online on a smartphone using the constrained nonlinear optimization, which is the main contribution. The constrained nonlinear optimization considers all the necessary bounds on the model parameters and the map of the space when constructing the path loss models. The obtained beacons’ locations are determined fairly accurately (with an average error of 0.5 m) and the models fit the measurements well, although they contain a lot of noise. From the obtained radiolocation results, it can be seen that the proposed PSO-based localization algorithm (with an average error of 0.8 m) combined with online constructed models can meet most localization requirements in smartphone applications. The developed system for automated data collection and online modeling on a smartphone has proven to be very useful as it can greatly simplify and speed up the installation of the Bluetooth network, especially in wide-area facilities such as airports, hospitals, museums etc., where the configuration time can be reduced by more than 90%. Further development of the system will focus on the more sophisticated fusion of the radiolocation and inertial system.
